# Draft Genome Sequence of Desulfurobacterium thermolithotrophum Strain HR11, a Novel Thermophilic Autotrophic Subspecies from a Deep-Sea Hydrothermal Vent

**DOI:** 10.1128/MRA.00167-20

**Published:** 2020-03-26

**Authors:** James F. Holden, Collin P. Bardwell, Srishti Kashyap

**Affiliations:** a Department of Microbiology, University of Massachusetts, Amherst, Massachusetts, USA; Portland State University

## Abstract

*Desulfurobacterium* sp. strain HR11 was isolated from a hydrothermal vent on the Juan de Fuca Ridge. We present the 1.55-Mb genome sequence of HR11, which contains 1,624 putative protein-coding sequences. Overall genome relatedness index analyses indicate that HR11 is a novel subspecies of D. thermolithotrophum.

## ANNOUNCEMENT

*Desulfurobacterium* sp. strain HR11 is a thermophilic autotroph isolated from low-temperature (19°C) hydrothermal vent fluid collected from the Endeavour Segment of the Juan de Fuca Ridge in the northeastern Pacific Ocean (1). Its 16S rRNA gene sequence is 99.3% identical to that of Desulfurobacterium thermolithotrophum BSA^T^, isolated from a hydrothermal vent on the Mid-Atlantic Ridge (2), which is above the 98.7% cutoff value for a novel species (3). However, unlike BSA, HR11 reduced nitrate, could not reduce sulfite, and grew more rapidly than BSA (Table 1). It also had 16S rRNA gene sequence and phenotypic differences compared with other *Desulfurobacterium* species (Table 1). Therefore, to determine if it is a novel species, the genome of HR11 was sequenced, and overall genome relatedness index (OGRI) analyses (3) were performed in comparison with BSA.

**TABLE 1:** Characteristics of Desulfurobacterium thermolithotrophum strain HR11 and related members of the genus

Characteristic	Data for strain^*a*^:					
	1	2	3	4	5	6
Cell shape	Short rod	Straight rod	Straight to curved rod	Straight to curved rod	Straight to curved rod	Straight rod
Length (μm)	1–2	1–2	0.9–3.5	0.9–2.2	1–2	2.5–3.5
Width (μm)	0.5–1	0.4–0.5	0.4–0.7	0.4–0.6	0.4–0.5	0.4–0.5
Temp (°C)	40–77 (72–75)	40–75 (70)	50–70 (60–65)	40–75 (65)	55–85 (75)	50–80 (70–75)
pH	5–8.5 (6–7)	4.4–8 (6)	5–7.5 (6)	5–8 (6)	5.5–7.5 (6)	5.5–7 (6)
NaCl (%)	1–5 (3–4)	1–4.6 (2.3)	2–4 (3)	1–4.5 (2.5)	1.5–5 (3)	1.5–5 (3)
Doubling time (min)	26	135	75	ND	ND	ND
Flagellation	Monopolar	Monopolar	Bipolar	Monopolar	Monopolar	Monopolar
16S rRNA gene identity (%)	100	99.3	97.4	94.3	94.8	95.2
G+C content (mol%)	34.7	36	37	38.3	42	41
Electron acceptor						
S_2_O_3_^2−^	+	+	−	+	+	+
S°	+	+	+	+	+	−
SO_3_^2−^	−	+	−	−	−	−
NO_3_−	+	−	+	+	+	−

a Optimal conditions are shown in parentheses. 1, *D. thermolithotrophum* HR11 (1); 2, *D. thermolithotrophum* BSA^T^ (2); 3, *D. crinifex* NE1206^T^ (4); 4, *D. indicum* K6013^T^ (5); 5, *D. pacificum* SL17^T^ (6); 6, *D. atlanticum* SL22^T^ (6); ND, not determined.

HR11 was grown as previously described (1), and its genomic DNA was extracted and purified using a Wizard genomic DNA purification kit (Promega, Madison, WI, USA) per the manufacturer’s protocol. Library construction was performed using a NexteraXT DNA library prep kit (Illumina, USA) per the manufacturer’s protocol. Both library construction and sequencing were performed by GENEWIZ (South Plainfield, NJ, USA). The DNA was sequenced using a MiSeq instrument (Illumina, USA) with 2 × 150-bp chemistry, generating a total of 6,937,786 raw paired-end reads. Default parameters were used for all software analyses. Trimmomatic version 0.36 (7) was used to trim the last 8 bp of each sequence and regions with low quality (Q) scores (Q < 30). The resulting paired-end sequences were then assembled using the SPAdes genome assembler version 3.10 (8), resulting in 41 high-quality contigs, with an *N*_50_ value of 77,485 bp and a maximum contig length of 173,616 bp. The assembled HR11 genome was 1,548,458 bp long, with a G+C content of 34.74%. Open reading frames (ORFs) were identified using EMBOSS tools (9) and annotated using Diamond BLASTp (10), resulting in 1,624 protein-coding genes. Five rRNA genes (3 copies of the 5S rRNA gene, 1 copy each of 16S and 23S rRNA genes) were identified using RNAmmer version 1.2 (11), and 39 tRNA genes were identified using tRNAscan-SE version 2.0 (12). Using PATRIC (13), the genome is 97.4% complete relative to the complete BSA genome sequence and contains 254 protein-encoding sequences (CDSs), including 45 contiguous CDSs that include CRISPR-Cas genes, that are absent from BSA.

For OGRI analyses, the BLAST-based average nucleotide identity (ANI) score was calculated using the JSpeciesWS program, version 3.2.2 (14). Genome-to-genome direct comparison (GGDC) analyses were performed using all three equations in the GGDC program, version 2.1 (15). Forty marker proteins defined in the species identification (SpecI) program (16) were manually compared using BLAST-P. The ANI score was 95.9%, the GGDC scores were 82%, 67%, and 82%, and the SpecI score was 98.5%, which were at or above their respective cutoff values for species determination (3, 16). However, the genomic and phenotypic differences between strain HR11 and *D. thermolithotrophum* BSA and the other *Desulfurobacterium* species (Table 1) suggest naming HR11 a novel subspecies of *D. thermolithotrophum*.

### Data availability.

This whole-genome shotgun project was deposited at DDBJ/ENA/GenBank under the accession number WSXR00000000. The version described in this paper is version number WSXR01000000. The raw reads were deposited in the Sequence Read Archive under run number SRR10619028 and BioProject number PRJNA580254.
